# Supplementary material for: Chromothripsis during telomere crisis is independent of NHEJ, and consistent with a replicative origin
Source: Genome Res. 2019 May;29(5):737–49. doi: 10.1101/gr.240705.118 (PMC6499312; doi:10.1101/gr.240705.118)
Supplement: Supplemental Material [file supp_gr.240705.118_Supplemental_file_1.zip › contigs/annotated_contigs/DB37/contig.2.DB37_length_289_mean_cov_2.07612456747.docx]

**DB37_length_289_mean_cov_2.07612456747**

```
GCTGCACTTGTAGTGTCAGGGTTATCAATATCCGTGGCTCTT|GACCCTGTTTTTCATACAGTGAGCACTCTTGAACCCAGAGAAGGTG
 >chr16:78675271-78675313 + E=6e-14         >chr16:78667938-78668185 + E=8e-135
CAGGTTGGAGATGCAGCTATATGACTCTGTACCAGAGAGAGACCTTTTTGTACATTTGGAATAAAAATTTTTAGCATTAGAGATTAGCT

TTTTTGTGTCTAAATACAAATGTCCAGGAGAATAGGAATGCCATAGTGGTGAGCTAAGAGCAGGCAGTTAGTTAAGTGTATGACAAGTA

TCTCATTATAAATGGATGCTGAA
```
